# To: Efficacy of melatonin in decreasing the incidence of delirium in critically ill adults: a randomized controlled trial

**DOI:** 10.62675/2965-2774.20250387

**Published:** 2025-08-18

**Authors:** Josef Finsterer

**Affiliations:** 1 Neurology and Neurophysiology Center Department of Neurology Vienna Austria Department of Neurology, Neurology and Neurophysiology Center - Vienna, Austria.

**To the Editor**

We were interested to read the article by Banyobadhyay et al. on a randomized controlled trial on the effect of enteral melatonin (3mg/d) on the prevention of *delirium* in 54 intensive care unit (ICU) patients on day 1, 3 and 7 of hospitalization, conducted between January 2020 and October 2020.^(1)^ Melatonin was found to be no more effective than standard treatment in preventing the onset of *delirium* within the first week of ICU stay.^(1)^ The study is convincing, but some points should be discussed.

The first point is that the etiology of *delirium* was not included in the evaluation.^(1)^ Since *delirium* is multicausal,^(2)^ the effect of melatonin may be highly dependent on the underlying cause of *delirium*. We should know how many of the included patients developed *delirium* due to cerebral disease, new onset psychiatric illness, comedications or comorbidities.

The second problem is that only coma was an exclusion criterion, not sopor or somnolence.^(1)^ How was it possible to study soporous or somnolent patients with the Confusion Assessment Method in the ICU (CAM-ICU)? Patients with impaired consciousness, regardless of degree, may answer the CAM-ICU questions incorrectly. In general, patients with impaired consciousness should have been excluded from the study. The inclusion of soporific or somnolent patients can falsify the results.

The third point is that apparently agitated patients who were at risk of self-extubation or weaning failure were given intravenous haloperidol,^(1)^ As haloperidol is a potent antidelirans, patients who had received haloperidol should have been excluded from the study.

The fourth issue is that the included patients were not systematically seen by a psychiatrist, but only by the intensivist who performed the CAM-ICU.^(1)^ As *delirium* is a psychiatric diagnosis (ICD code: F05),^(3)^ it would have been mandatory to have all patients assessed by a psychiatrist, also to assess the difference in the diagnosis of *delirium* by the CAM-ICU and by the psychiatrist. Involving the psychiatrist only for patients who remained agitated despite haloperidol is not sufficient.

The fifth point is that cerebral imaging or electroencephalogram (EEG) recordings were not systematically performed in the included patients. Since *delirium* can be a manifestation of cerebral disease^(4)^ or epileptic activity, it would have been imperative not only to perform a cerebral magnetic resonance imaging but also to record EEGs from each included patient. In particular, hyperactive *delirium* can easily be confused with generalized clonic seizures.

The sixth point is that the CAM ICU might miss patients with hypoactive *delirium*. We should know whether some of the included patients also had hypoactive *delirium* and how this was diagnosed. Hypoactive *delirium* would have been another reason why all patients should have been evaluated by a psychiatrist and neurologist, with EEG, imaging and possibly cerebrospinal fluid testing.

Overall, it can be said that this interesting study has limitations that put the results and their interpretation into perspective. Addressing these limitations could strengthen the conclusions and corroborate the study's message. Before the effect of melatonin as an anti-delirans in ICU patients can be evaluated, the etiology of *delirium* should be uncovered.
